# Cervical microbiota in women with cervical intra-epithelial neoplasia, prior to and after local excisional treatment, a Norwegian cohort study

**DOI:** 10.1186/s12905-019-0727-0

**Published:** 2019-02-06

**Authors:** Johanna Wiik, Verena Sengpiel, Maria Kyrgiou, Staffan Nilsson, Anita Mitra, Tom Tanbo, Christine Monceyron Jonassen, Tone Møller Tannæs, Katrine Sjøborg

**Affiliations:** 1 Department of Gynecology and Obstetrics, Østfold Hospital Trust, Kalnes, Norway; 2 Department of Obstetrics and Gynecology, Sahlgrenska Academy, Gothenburg University, Gothenburg, Sweden; 3 Department of Obstetrics and Gynecology, Sahlgrenska University Hospital, Gothenburg, Sweden; 4 Department of Surgery & Cancer, IRDB, Faculty of Medicine, Imperial College, London, W12 0NN UK; 5 West London Gynaecological Cancer Center, Queen Charlotte’s & Chelsea – Hammersmith Hospital, Imperial Healthcare NHS Trust, London, W12 0HS UK; 6 Department of Mathematical Sciences, Chalmers University of Technology, Gothenburg, Sweden; 7 Department of Pathology and Genetics, Institute of Biomedicine, University of Gothenburg, Gothenburg, Sweden; 8 Department of Reproductive Medicine, Oslo University Hospital, Oslo and Institute of Clinical Medicine, University of Oslo, Oslo, Norway; 9 Center for Laboratory Medicine, Østfold Hospital Trust, Kalnes, Norway; 10 Department of Clinical Molecular Biology (EpiGen), Division of Medicine, Akershus University Hospital and University of Oslo, Oslo, Norway

**Keywords:** Vaginal microbiota, HPV, Human papillomavirus, Lactobacillus, Cervical Intraepithelial Neoplasia, CIN, LEEP

## Abstract

**Background:**

Local treatment for cervical intraepithelial neoplasia (CIN) by Loop Electrosurgical Excision Procedure (LEEP) has been correlated with reproductive morbidity, while the cervicovaginal microbiota is also known to affect the risk of preterm delivery. CIN and treatment by LEEP might change the cervical microbiota. The main aim of this study was to describe the cervical microbiota before and after LEEP and assess its associaton with cone depth and HPV persistence. Further, we aimed to compare the microbiota to references with normal cervical cytology.

**Methods:**

Between 2005 and 2007, we prospectively identified 89 women planned for LEEP in a Norwegian hospital and recruited 100 references with a normal cervical cytology. Endocervical swabs were collected prior to treatment and at six (*n* = 77) and 12 months (*n* = 72) post LEEP for bacterial culture and PCR, and post LEEP for DNA testing for human papillomavirus (HPV). We compared the cervical microbiota composition before and after treatment and between women planned for LEEP vs references.

**Results:**

There was a reduction in the number of non-*Lactobacillus* bacterial species six and 12 months after LEEP compared to before treatment and a tendency towards a concomitant increase in *Lactobacillus*. No association between the detection of cervical bacteria, HPV persistence or cone depth was found.

Women planned for LEEP carried significantly more *Bacteroides* spp., *Gardnerella vaginalis, Mycoplasma hominis* and *Ureaplasma parvum* as well as a greater number of bacterial species than the references.

**Conclusions:**

Local excisional treatment appears to alter the cervical microbiota towards a less diverse microbiota. Women with CIN have a more diverse cervical microbiota compared to women with normal cervical cytology.

**Electronic supplementary material:**

The online version of this article (10.1186/s12905-019-0727-0) contains supplementary material, which is available to authorized users.

## Key message

Local excisional treatment appears to alter the cervical microbiota towards a less diverse microbiota. Women with CIN have a more diverse microbiota than women without CIN.

## Background

Persistent genital infection with high-risk human papillomavirus (HPV) is causally associated with cervical cancer and its precursors (cervical intra-epithelial neoplasia; CIN) [1]. A number of biological and environmental co-factors may promote malignant transformation, whilst others are known to be protective. There is emerging evidence indicating that the cervicovaginal microbiota is important for HPV persistence and development of premalignant lesions. *Chlamydia trachomatis, Trichomonas vaginalis* and herpes simplex virus*-*2 have been associated with HPV-related cervical neoplastic lesions and carcinoma [2].

Health in the female reproductive tract is commonly associated with low microbial diversity and dominance by one or a few species of *Lactobacillus* [3]. Dysbiosis is a term used to refer to an altered microbiota. In the lower female reproductive tract this means a lack of *Lactobacillus* with subsequent overgrowth of anaerobes, in the vagina also referred to as bacterial vaginosis (BV)*.* BV has been associated with higher prevalence and persistence of HPV infection and with development of CIN [4–6]. Recent studies using next generation sequencing techniques suggest that increased diversity of the vaginal microbiota combined with reduced relative abundance of *Lactobacillus* spp. is involved in HPV acquisition and persistence, and in the development of CIN and cancer [7–11].

Loop Electrosurgical Excision Procedure (LEEP) is the most commonly used treatment technique for CIN. The majority of the women treated are young and have not started or completed their reproductive career. Local excisional treatment has been associated with an increased risk of preterm delivery (PTD) in subsequent pregnancies [12–14]. The underlying mechanism of how excisional treatment increases the risk of PTD remains unclear. Lack of mechanical support may partly explain the increase in risk, evidenced by the impact that the depth of the excision has on the magnitude of risk [14, 15]. An ascending bacterial infection from the lower genital tract into the uterine cavity is a possible pathomechanism for PTD [16, 17] and CIN treatment may hypothetically alter the cervicovaginal microenvironment and increase susceptibility to ascending infections when women become pregnant. Cervical excisional treatment has been associated with an increased risk of chorioamnionitis [14] but one retrospecitve study found no increased risk for vaginal infections in pregnant women with a history of LEEP compared to women with no history of LEEP [18]. How LEEP influences the genital microbiota has only recently been studied by Zhang et al. who found a decrease of cervical microbial diversity and an increase of *Lactobacillus* in a small non-pregnant population three months after LEEP [19].

The main aim of this prospective observational study was to compare the cervical microbiota in women with CIN before and after LEEP and to assess whether cone depth and/or HPV persistence affect bacterial composition post-treatment. An additional aim was to compare the cervical microbiota in women with CIN to healthy reference women with normal cervical cytology.

## Methods

### Study population

**Fig. 1:**
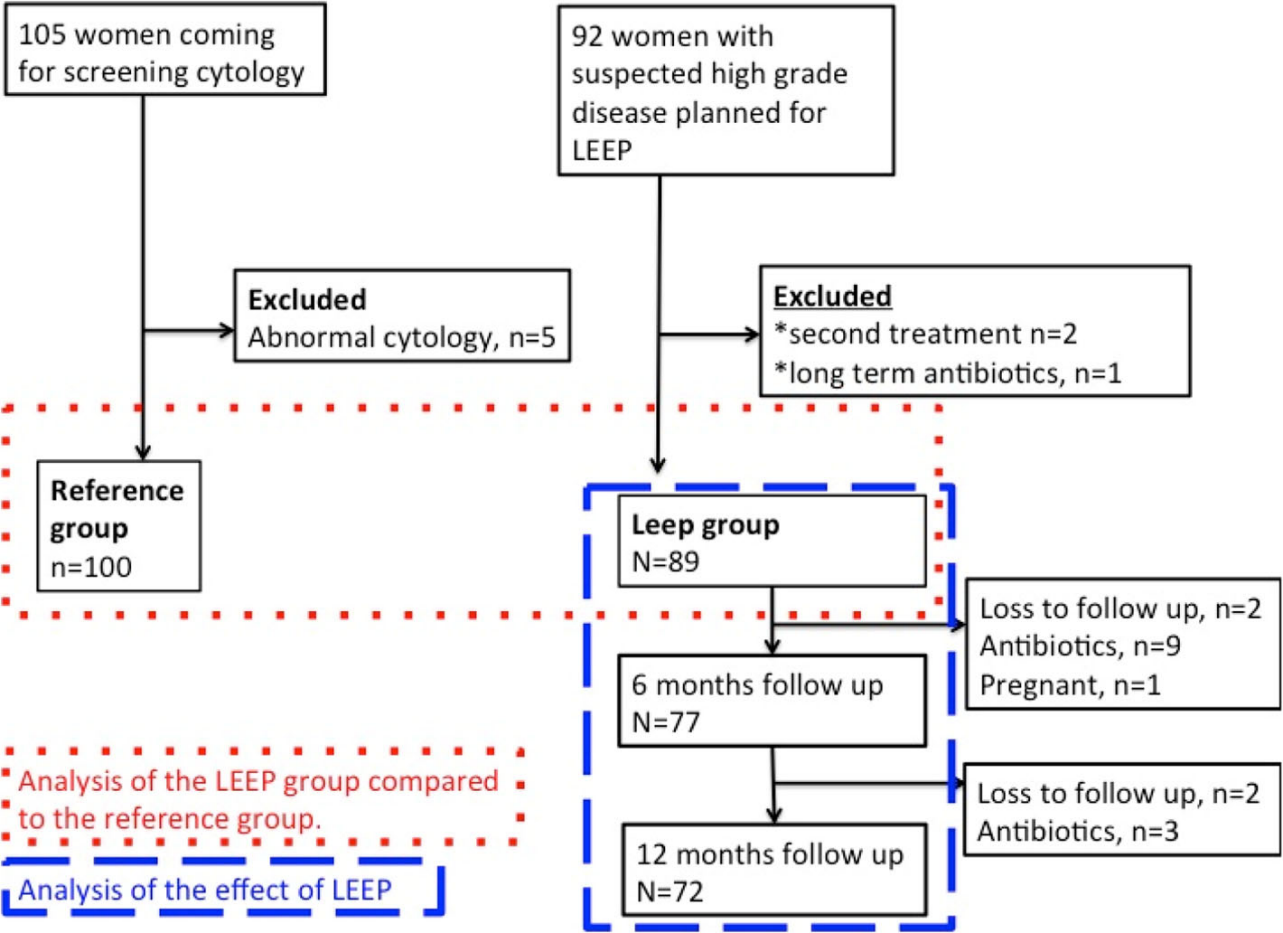
Flowchart of formation of the study groups and follow up

We prospectively identified all women referred for investigation of cervical dysplasia at Østfold Hospital Trust, Health Region East, Norway between October 2005 and February 2007. Women planned for their first LEEP for suspected high-grade disease were eligible (Fig. 1). Women on long-term antibiotics were excluded leaving 89 eligible women in the case group, termed the “LEEP-group”. Of these, 81 women were treated for CIN3, six for CIN2 and two for persistent CIN1. Women requiring antibiotics after LEEP and pregnant women were excluded in the follow-up analysis. Two women were lost to follow up at six months and four women at 12 months leaving 77 women for the follow up analysis at six months and 72 women at 12 months.

A population of 100 healthy references, defined by the presence of normal cytology, was recruited at a private gynecological practice, and termed the “reference group” (Fig. 1).

A detailed history was taken by the consulting clinician to include sociodemographical data, including year of birth, education level, marital status, smoking habits and medical history including use of recent antibiotics and contraception.

### Sample collection

#### Microbiota

With sterile speculum examination, serial Liquid Based Cytology (LBC) and five endocervical bacterial swabs were collected prior to treatment and at six and 12 months post-treatment as well as at baseline for the reference population. One swab was transported on Stuart medium for cultivation of anaerobic and aerobic bacteria on blood and lactose agar plates. Growth results were reported as growth or no growth for the following bacteria; *Bacteroides* spp., *Escherichia coli*, *Gardnerella vaginalis*, *Streptococcus* spp. and *Lactobacillus* spp. Other bacterial species detected at cultivation were also reported. The four remaining swabs were transported in Copan Universal Transport Medium (UTM-RT) for detection of *Chlamydia trachomatis*, using the COBAS TaqMan 48 CT test (Roche Molecular Diagnostics, Pleasanton, CA), and for detection of *Mycoplasma hominis*, *Ureaplasma urealyticum* and *Ureaplasma parvum*. DNA was extracted from the swabs using the BioRobot M48 (Qiagen, Hilden, Germany) and the Mag Attract DNA bact protocol. Amplification and detection of *U. parvum*, *U. urealyticum* and *M. hominis* were performed using the ABI Prism 7900HT Sequence Detection System (Applied Biosystems, Foster city, CA) [20, 21].

In every woman all bacterial species found were recorded. All women that had at least one type of one non-*Lactobacillus* bacteria were included in a constructed group called“ any non-*Lactobacillus*”.

#### HPV tests and histology

Cervical LBC specimens were collected with a Cytobrush Plus (Medscan Medical AB, Sweden) and transferred to PreservCyt medium (item no 0234005, Cytyc Corporation, Marlborough, USA) in the follow-up samples of the treated population. Nucleic acid extraction and high-risk HPV DNA testing (Amplicor HPV test, Roche Diagnostics Switzerland) was performed as previously described [22]. All biopsy and cone histological specimens were re-evaluted blindly by the same experienced pathologist and diagnosed according to WHO classification [23]. The cone depth was documented in millimeters (mm). The specimen with the most severe lesion was chosen for diagnosis.

### Statistical analyses

Background characteristics of sociodemographical data and contraception in the reference group and the LEEP group were compared using chi-square or independent t-tests.

The number of different non-*Lactobacillus* species at each assessment was recorded. The mean number of non-*Lactobacillus* before and after LEEP was compared with paired t-test (at six or 12 months). The change in mean number of non-*Lactobacillus* after LEEP was compared between women below 46 years and women 46 years and above with independent t-test. This change in mean number of non-*Lactobacillus* after six and 12 months follow up was also compared between women without hormonal contraceptive and women with hormonal contraceptive and between women in a relationship (married/cohabiting) and single women. To study if LEEP resulted in a change of different bacterial species in each individual we focused on the women with a change in detection of bacteria after LEEP compared to before LEEP (those who either lost or aquired bacteria after LEEP compared to before LEEP). We compared the number of women who lost a specific species of bacteria that were present before LEEP to the number of women that aquired that particular bacteria species after LEEP. This was performed using paired data from before LEEP and from follow up (at six or 12 months) with McNemar test.

HPV DNA status post-treatment for assessment of persistence of HPV was studied. The mean number of non-*Lactobacillus* was compared between HPV negative and HPV positive women with independent t-test at six and 12 months follow up. Difference in detection of *Lactobacillus* and the constructed group any non-*Lactobacillus* according to HPV DNA positivity after LEEP was analysed with Fisher’s exact test. The mean aquisition or loss of detectable non-*Lactobacillus* bacterial species at six and 12 months follow up compared to before treatment was analysed in relation to HPV-DNA status after LEEP using independent t-test.

We compared the cone depth between women testing positive or negative for HPV DNA at both six and 12 months with independent t-test. We further assessed whether change in the microbiota (of the number of different species of non-*Lactobacillus*) at six and 12 months after treatment was correlated to the cone depth using the Pearson Correlation test.

The mean number of detected bacterial species in the reference group and the LEEP group before treatment was compared using an independent t-test. The differences in individual bacterial species detected and the constructed broader group“ any non-*Lactobacillus*” were compared between the reference group and the LEEP group before treatment and at six and 12 months follow up using Fishers exact test and logistic regression adjusted for age (< 46, 46–55, > 55 years), marital status (living together (married/cohabiting) or living alone (single)), smoking (none, 1–10/day, > 10/day) and use of hormonal contraception (yes or no).

Statistical analyses were performed for the whole population and in a subgroup including only women below the age of 46 (assumed premenopausal).

For all statistical tests a *p*-value < 0.05 was accepted as statistically significant. Statistical analyses were performed using IBM SPSS software, version 24.0.

## Results

Women planned for treatment were significantly younger than the healthy references, more likely to use contraception, to smoke and less likely to be married (Table 1). No woman reported a change in contraceptive use at follow up.

**Table 1:** Characteristics of participants by groups

Characteristics	LEEP group *N*=89		Reference group *N*=100		p
Age, years median (IQR)	36 (31-42)		47 (38-56)		<0.001^1^
	n	%	n	%	
Age < 46 years	75	84	48	48	<0.001^2^
Smoking					<0.001^2^
None	48	54	82	83	
1-10 / day	8	9	8	8	
>10 / day	33	37	9	9	
Unknown	0		1		
Contraceptive use					<0.001^2^
None	39	44	73	73	
Hormonal IUD	20	22	14	14	
Copper IUD	7	8	6	6	
Contraceptive pills/plaster	17	19	3	3	
Condom	0	0	2	2	
Vaginal Hormonal Ring	1	1	0	0	
Gestagen injection	2	2	2	2	
Sterilisation	3	3	0	0	
Hormonal contraceptive	40	45	19	19	<0.001^2^
Marital status					<0.001^2^
Married/cohabiting	49	55	79	79	
Single/living alone	40	45	21	21	
Education					0.09^2^
9-14 years of education	60	68	56	56	
>14 years of education	28	32	44	44	
Unknown	1		0		

^1^Independent t-test

^2^Chisquare test

Abbreviations *IUD* intrauterine device, *LEEP* Loop Electrosurgical Excisional Procedure, *n* number

The bacteria composition in the LEEP group before and after LEEP as well as in the reference group are described in Table 2. *Ureaplasma parvum* was the most frequently detected bacteria in the LEEP group before treatment (46%), followed by *Lactobacillus spp*. (36%) and *Gardnerella vaginalis* (32%). *Lactobacillus spp.* was the most frequently detected bacteria in the reference group (39%) followed by *Ureaplasma parvum* (16%).

**Table 2:** The cervical microbiota in the LEEP group and Reference group, before and after treatment

	LEEP group *N* = 89		6 m follow-up LEEP group *N* = 77		12 m follow-up LEEP group *N* = 72		Reference group *N* = 100	
Bacteria	n	%	n	%	n	%	n	%
*Bacteroides* spp.	7	8	2	3	3	4	0	0
*Chlamydia trachomatis*	3	3	0	0	0	0	1	1
*Escherichia.coli*	4	5	2	3	2	3	9	9
*Gardnerella vaginalis*	28	32	21	27	20	28	9	9
*Mycoplasma hominis*	17	19	10	13	8	11	3	3
*Streptococcus* spp.	12	14	2	3	3	4	10	10
*Ureaplasma parvum*	41	46	25	32	26	36	16	16
*Ureaplasma urealyticum*	9	10	3	4	4	6	3	3
*Difteroides*	2	2	0	0	0	0	1	1
*Fusobacteria*	1	1	0	0	0	0	0	0
*Klebsiella*	0	0	2	3	0	0	2	2
*Prevotella*	1	1	0	0	0	0	0	0
*Stafylococcus aureus*	0	0	1	1	0	0	0	0
*Lactobacillus* spp.	32	36	36	47	39	54	39	39
any non-*Lactobacillus*	59	66	44	57	39	54	41	41

Abbreviations: *LEEP* Loop Electrosurgical Excisional Procedure, *m* months, *M* mycoplasma, *N* numbers

### Comparison of microbiota in women before and after LEEP

The individual bacterial species detected before and after LEEP were largely similar. The number of non-*Lactobacillus* bacterial species decreased after treatment (mean number: before treatment vs after 6 m 1.29 vs 0.88*, p = 0.004*, before treatment vs after 12 m 1.22 vs 0.92, *p =* 0.046). No significant difference in the change of non-*Lactobacillus* species numbers after LEEP was found when comparing women below 46 years to women 46 years and above, women without hormonal contraceptive to women with hormonal contraceptive or women in a relationship (married/cohabiting) to single women (data not shown).

When analysing individual bacterial species more women had cleared than aquired *Streptococcus* spp. (*p* = 0.04) and *Ureaplasma parvum* (*p* = 0.04) at 6 months follow up. These differences were no longer significant at the 12 months follow up (Table 3).

**Table 3:** The cervical microbiota six and 12 months after LEEP compared to before LEEP

	6 months after LEEP (*N* = 77)			12 months after LEEP (*N* = 72)		
Bacteria	Pos- > Neg n	Neg.- > Pos n	p^1^	Pos- > Neg n	Neg- > Pos n	p^1^
*Bacteroides* spp	5	1	0.22	5	3	0.73
*Escherichia.coli*	2	1	1,00	3	1	0.63
*Gardnerella vaginalis*	8	8	1.00	7	7	1.00
*Mycoplasma hominis*	5	1	0.22	5	2	0.45
*Streptococcus* spp	8	1	0.04	8	3	0.23
*Ureaplasma parvum*	15	5	0.04	10	5	0.30
*Ureaplasma urealyticum*	5	1	0.22	5	3	0.73
*Lactobacillus* spp	12	17	0.46	8	18	0.08

^1^Mc Nemar test

Abbreviations; *LEEP* Loop Electrosurgical Excisional Procedure, *N* numbers, *neg* negative, *pos* positive

There was a tendency towards an increase in *Lactobacillus* spp. after 12 months, although the difference was not significant. The subgroup analysis in women below the age of 46 for change of individual bacterial species showed no apparent difference but the sample size was small (data not shown).

### HPV status and microbiota in women after LEEP

At six months, 24 of 77 (31%) and at 12 months 24 of 72 (33%) treated women had a positive HPV DNA test. The mean number of non-*Lactobacillus* in the HPV pos and the HPV neg group at 6 months (0.79 vs 0.92, *p* = 0.57) and at 12 months (1.17 vs 0.79, *p* = 0.19) did not differ. No apparent difference was seen when restricting the analysis to women below 46 years of age. No difference in the detection of *Lactobacillus* or the occurrence of the any non-*Lactobacillus* group according to HPV DNA positivity after LEEP was found (Table 4).

**Table 4:** HPV status and cervical microbiota after LEEP

	6 months after LEEP, (*N* = 77)					12 months after LEEP, (*N* = 72)				
	HPV-DNA pos (*N* = 24)		HPV–DNA neg (*N* = 53)			HPV-DNA pos (N = 24)		HPV–DNA neg (*N* = 48)		
Bacteria	Pos Bact		Pos Bact		p^1^	Pos Bact		Pos Bact		p^1^
	n	%	n	%		n	%	n	%	
*Lactobacillus* spp	13	54	23	43	0.46	12	50	27	56	0.63
any non-*Lactobacillus*	14	58	30	57	1.00	13	54	26	54	1.00

^1^Fisher’s exact test

Abbreviations; *bact* bacteria, *DNA* DeoxyriboNucleic Acid, *HPV* Human Papilloma Virus, *LEEP* Loop Electrosurgical Excisional Procedure, *N* numbers, *neg* negative, *pos* positive

The mean loss of detectable bacterial species after LEEP was not associated with HPV status post treatment (mean loss after 6 months HPV pos 0.63 vs HPV neg 0.30 *p =* 0.28; and after 12 months HPV pos 0.29 vs HPV neg 0.31 *p* = 0.95).

### Cone depth and microbiota in women before and after LEEP

The cone depth of the 77 treated cases varied between 10 and 25 mm (mean 18.0 mm). The mean cone depth was similar for women testing positive or negative for HPV DNA at six months (pos 17.9, neg 18.0, *p =* 0.9) and 12 months (pos 17.6, neg 18.2 *p =* 0.6). There was no correlation between cone depth and change in the number of non-*Lactobacillus* at follow up compared to before treatment (data not shown).

### Comparisons of microbiota in women planned for treatment versus healthy references

**Fig. 2:**
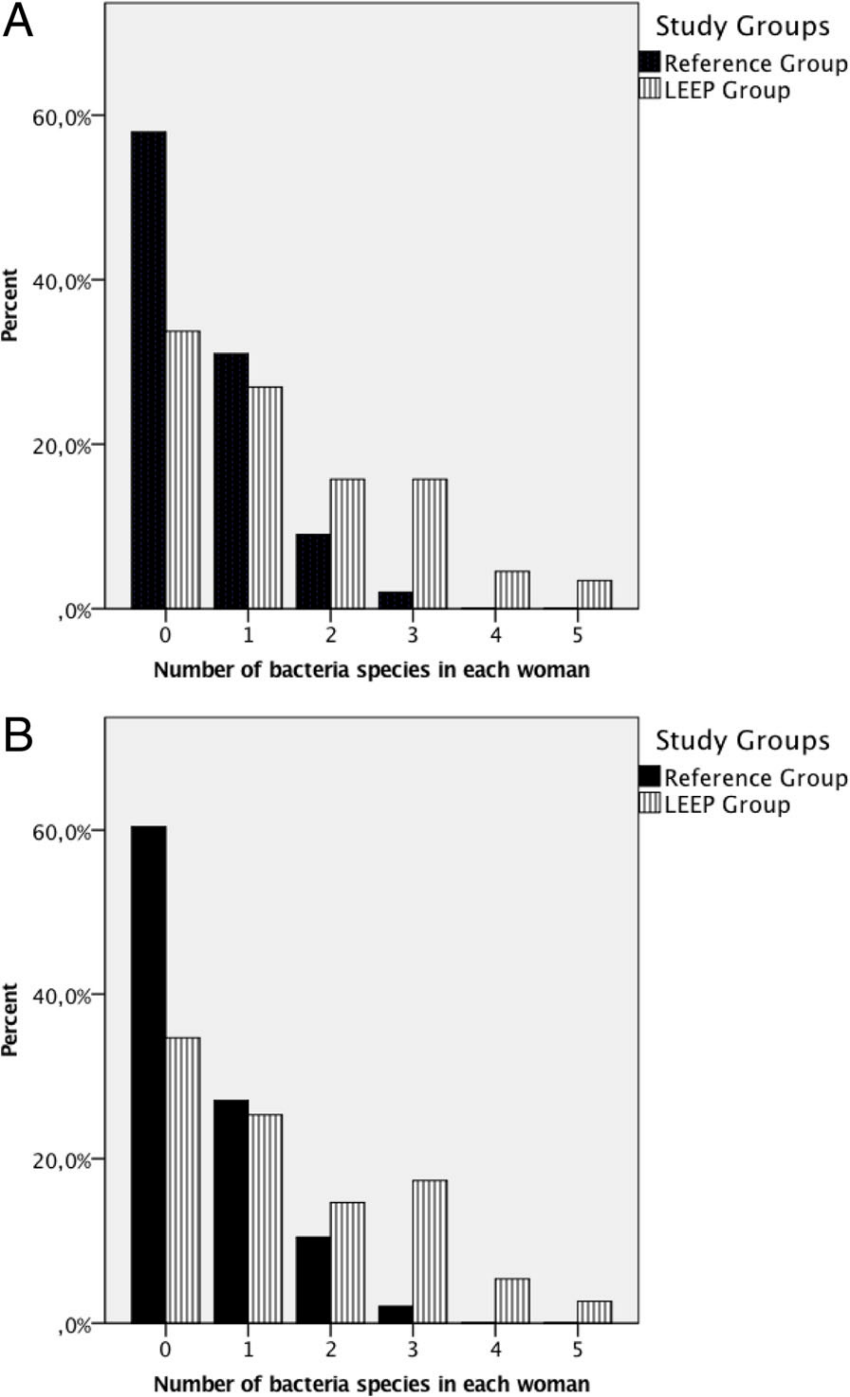
Number of non-*Lactobacillus* bacteria identified in culture or PCR (range: 0–5) for women planned for LEEP as opposed to references. **a**) All women **b**) Women aged <46 years

Women in the LEEP group had more non-*Lactobacillus* compared to the references (mean number of non-*Lactobacillus* 1.40 vs 0.55, *p* = < 0.001), Fig. 2. The result was similar for women below and above 46 years old (data not shown). Also when including only those positive for any non-*Lactobacillus* bacterial specie, the women in the LEEP group had a significantly higher bacterial diversity before treatment as compared to the reference group (mean number of non-*Lactobacillus* 2.12 vs 1.32, *p* = < 0.001). *Bacteroides* spp., *Gardnerella vaginalis, Mycoplasma hominis* and *Ureaplasma parvum* were more often detected in the LEEP group before treatment compared to the reference group (Table 5). There was no difference in the presence of *Lactobacillus* spp*.* (*p* = 0.76) (Table 5).

**Table 5:** The cervical microbiota in the LEEP group and the Reference group

	LEEP group *N* = 89		Reference group *N* = 100		Unadjusted^1^			Adjusted^2^		
Bacteria	n	%	n	%	OR	CI	p	OR	CI	p
*Bacteroides* spp	7	8	0	0	**∞**		0.005	**∞**		0.008
*Chlamydia trachomatis*	3	3	1	1	3.5	0.4–33.8	0.34	2.3	0.2–26.0	0.52
*Escherichia.coli*	4	4	9	9	0.5	0.1–1.6	0.26	0.3	0.1–1.4	0.12
*Gardnerella vaginalis*	28	32	9	9	4.6	2.1–10.5	<.001	3.5	1.4–9.0	0.009
*Mycoplasma hominis*	17	19	3	3	7.6	2.2–27.0	0.001	2.4	0.6–9.5	0.21
*Streptococcus* spp	12	14	10	10	1.4	0.6–3.4	0.50	1.9	0.7–5.5	0.24
*Ureaplasma parvum*	41	46	16	16	4.5	2.3–8.8	<.001	3.9	1.6–9.3	0.002
*Ureaplasma urealyticum*	9	10	3	3	3.6	1.0–13.9	0.07	2.2	0.5–10.5	0.32
*Lactobacillus* spp	32	36	39	39	0.9	0.5–1.6	0.76	0.8	0.4–1.7	0.58
any non-*Lactobacillus*	59	66	41	41	2.8	1.6–5.1	0.001	2.2	1.1–4.5	0.03

^1^Fisher’s exact test

^2^Logistic regression adjusted for Marital status, Hormonal Contraceptive Use, Smoking and Age. 188 cases analysed, 1 missing case in the reference group because of missing data on smoking

Abbreviations; *CI* Confidence Interval, *LEEP* Loop Electrosurgical Excisional Procedure, *N* numbers, *OR* Odds Ratio

The results were similar in the subgroup analysis of women below the age of 46 (Additional file 1: Table S1).

In order to assess whether the cervical bacteria changes after LEEP were a result of treatment rather than the removal of the disease, we compared the findings post-treatment at six and 12 months in the LEEP group to the healthy references. Women in the LEEP group had more non-*Lactobacillus* species at six and 12 months as compared to the references (mean number of non-*Lactobacillus* 0.88 and 0.92 vs 0.55), *p* = 0.009 and *p* = 0.01. For analyses of differences in detection of specific bacterial species see Additional file 1: Table S2 and Table S3.

## Discussion

In this study we found a reduction in the number of non-*Lactobacillus* bacterial species six and 12 months after LEEP compared to before treatment and a tendency towards a concomitant increase in *Lactobacillus*. The results were not significant for individual bacterial species, possibly due to the small sample size. No association between the detection of cervical bacteria and HPV persistence was found. The cone depth had no impact on the cervical microbiota or HPV status.

Our findings are in agreement with, to our knowledge, the only other study published on this topic so far. Zhang et al. recently published their results of comparing the cervical microbiota, studied with next generation sequencing (NGS) technique, before LEEP and at three months follow up in 26 women [19]. They reported a decrease of cervical microbial diversity after treatment, as we have also shown, but they also demonstrated a shift towards a *Lactobacillus iners* dominated microbiome. Due to the culture and PCR-based bacterial detection techniques used in our study, we were unable to describe the full taxonomy and different *Lactobacillus* species in the same way as Zhang et al.

In our additional analysis, we found a higher number of bacterial species in women with CIN prior to LEEP as opposed to references with a normal cytology, indicating that women with high-grade dysplasia harbour a more diverse cervical microbiota than the healthy female population. Our results are in agreement with earlier studies showing increased vaginal microbiota diversity and reduced levels of *Lactobacillus* spp. in association with preinvasive cervical disease states [6–9, 11].

The pathogenesis of the association between dysbiosis and development of CIN is unknown. It has earlier been suggested that dysbiosis may result in epithelial cell damage that potentially facilitates entry of HPV into the basal epithelial cells as well as creating an environment that promotes the viral life cycle, persistence of infection and ultimately development of dysplasia [7]. Levels of vaginal proinflammatory cytokines are higher in women with dysbiosis [24, 25], and chronic inflammation is a well-known factor in carcinogenesis of numerous tissues in the body [26].

Having CIN alone has been shown to confer a small increased risk of PTD [14, 27, 28] while excisional treatment increases this further. The frequency and severity of PTD seem to correlate to the cone depth [14]. The pathogenesises of these associations are still unknown. One potential mechanism could be alterations of the cervicovaginal microbiota. Previous studies have shown that women with spontaneous PTD and intact membranes often have intrauterine bacteria that include *M. hominis*, *Gardnerella vaginalis* and *Bacteroides* [29]. In our study of a non-pregnant cohort of women, these bacteria species were more frequently found in the cervix in the LEEP group than in the reference group. *U. Parvum,* that also has been associated with PTD [30], was also more frequently found in the LEEP group than in the reference group. It may be that the association between dysbiosis and CIN could partly explain the small increase in risk for PTD in pregnant women with untreated CIN. To answer this it would be interesting to study the cervical microbiota in untreated pregnant women with and without CIN. In our cohort of non-pregnant women local excisional treatment did not increase dysbiosis. Instead there was a tendency towards a reduction in the number of bacterial species with a further increase in *Lactobacillus* spp. Furthermore, although the cone depth correlates with the risk of PTD [14] this had no impact on the microbiota composition or HPV persistence rates in our non-pregnant cohort. These findings may support the hypothesis that it is more likely that mechanical factors rather than an altered cervical microbiota increase the risk for PTD after LEEP. However, our study was performed in a non-pregnant cohort and results may be different during pregnancy and parturition.

### Strengths and limitations

To our knowledge this is the second study, and the study with the largest sample size, that monitor changes in cervical microbiota before and after LEEP. This study includes follow up at two serial time points, including also a reference populaton of healthy individuals. However, there are some limitations in study design which have to be considered when interpreting the results: The history of recent sexual activity, the menopausal status and the use of postmenopausal hormone replacement therapy was unknown, and these factors may have an impact on the results. We performed subgroup analyses in women below the age of 46 (assumed premenopausal); the results were largely consistent with the total population although the sample size was smaller, facing the risk of type II-error, especially when studying specific bacteria species. The highest diversity and instability in microbiota is usually observed at the time of menstruation when oestrogens and progesterones are at their lowest [31]. The exact time of last menstruation was unknown but none of the samples were collected during menstruation.

Regarding the additional analysis, there were considerable differences in the characteristics between the LEEP and reference group, for example age. This may have affected the results as we know that several environmental and hormonal factors may affect the microbiota. Furthermore there was no follow-up in the reference group to compare any changes in microbiota composition over time. When planning similar studies in the future we recommend detailed collection of information about menopausal status, use of hormone replacement therapy, the exact time of menstruation, menstrual hygiene practices, use of vaginal douching, use of hormonal or barrier contraceptive products, sexual behaviour, number of sexual partners, history of recent sexual activity and use of antibiotics/probiotics (oral or topical) since these factors could affect the cervical microbiota.

## Conclusions

We observed a tendency of decreased bacterial diversity and a concomitant increase in *Lactobacillus* spp. six and 12 months after treatment, possibly reflecting the impact of the removal of the disease. These results support recent observations at three months follow up by Zhang et al. [19]. Further, this study supports earlier findings in the vaginal microbiota that women with HPV infection/ cervical dysplasia have a more diverse microbiota than women with normal cytology.

The sample sizes for some of the comparisons were small. Further, larger longitudinal studies with NGS technique in pre-menopausal women are needed to investigate the changes and stability of the microbiota after local excisional treatment. The analysis based on bacterial 16S rRNA used in NGS will permit an in-depth study of the vaginal microbial community structures to a level of detail not possible with standard culture-based microbiological techniques. Sampling should be expanded to include pregnant populations post-LEEP together with markers of inflammation to correlate results to clinical outcomes. Longitudinal studies are also necessary to confirm temporal relationships between cervicovaginal microbiota and HPV infection and development of dysplasia.

## Additional file

Additional file 1: **Table S1.** The cervical microbiota in the LEEP group and the Reference group in women aged <46 years. **Table S2.** The cervical microbiota six months post treatment in the LEEP group and the cervical microbiota in the Reference group. **Table S3.** The cervical microbiota 12 months post treatment in the LEEP group and the cervical microbiota in the Reference group. (DOC 88 kb)

## Abbreviations

BV: Bacterial Vaginosis
CIN: Cervical Intra-epithelial Neoplasia
DNA: DeoxyriboNucleic Acid
HPV: Human Papillomavirus
LEEP: Loop Electrosurgical Excision Procedure
NGS: Next Generation Sequencing
PTD: Preterm Delivery
