# Supplementary material for: Cervical microbiota in women with cervical intra-epithelial neoplasia, prior to and after local excisional treatment, a Norwegian cohort study
Source: BMC Womens Health. 2019 Feb 6;19:30. doi: 10.1186/s12905-019-0727-0 (PMC6364458; doi:10.1186/s12905-019-0727-0)
Supplement: Supplementary file 1 — Table S1. The cervical microbiota in the LEEP group and the Reference group in women aged <46 years. Table S2. The cervical microbiota six months post treatment in the LEEP group and the cervical microbiota in the Reference group. Table S3. The cervical microbiota 12 months post treatment in the LEEP group and the cervical microbiota in the Reference group. (DOC 88 kb) [file 12905_2019_727_MOESM1_ESM.doc]

**Additional file 1**

**Supporting information**

Table S1 The cervical microbiota in the LEEP group and the Reference group in women aged < 46 years.

|  | **LEEP**  **group**  **N=75** | | **Reference group**  **N=48** | | **Unadjusted^1^** | | | **Adjusted^2^** | | |
| --- | --- | --- | --- | --- | --- | --- | --- | --- | --- | --- |
| **Bacteria** | **n** | **%** | **n** | **%** | **OR** | **CI** | **p** | **OR** | **CI** | **p** |
| *Bacteroides* spp | 6 | 8 | 0 | 0 | ∞ |  | 0.08 | ∞ |  | 0.08 |
| *Chlamydia trachomatis* | 3 | 4 | 1 | 2 | 2.0 | 0.2-19.4 | 1.00 | 2.3 | 0.2-26.0 | 0.52 |
| *Escherichia.coli* | 4 | 5 | 5 | 10 | 0.5 | 0.1-1.9 | 0.31 | .5 | 0.1-2.1 | 0.31 |
| *Gardnerella vaginalis* | 24 | 32 | 4 | 8 | 5.2 | 1.7-16.1 | 0.002 | 4.0 | 1.2-13.3 | 0.02 |
| *Mycoplasma hominis* | 17 | 23 | 3 | 6 | 4.4 | 1.2-15.9 | 0.02 | 2.4 | 0.6-9.5 | 0.21 |
| *Streptococcus* spp | 9 | 12 | 3 | 6 | 2.1 | 0.5-8.0 | 0.36 | 1.5 | 0.3-6.6 | 0.62 |
| *Ureaplasma parvum* | 33 | 44 | 7 | 15 | 4.6 | 1.8-11.6 | 0.001 | 3.0 | 1.1-8.1 | 0.03 |
| *Ureaplasma urealyticum* | 8 | 11 | 2 | 4 | 2.8 | 0.6-13.5 | 0.31 | 2.1 | 0.4-11.6 | 0.41 |
| *Lactobacillus* spp | 27 | 36 | 22 | 46 | 0.7 | 0.3-1.4 | 0.35 | .7 | 0.3-1.7 | 0.44 |
|  | | | | | | | | | | |
| any non-*Lactobacillus* | 49 | 65 | 18 | 38 | 3.1 | 1.5-6.7 | 0.003 | 2.1 | 0.9-4.8 | 0.09 |

^1^ Fisher`s exact test

^2^ Logistic regression adjusted for Marital status, Hormonal Contraceptive, Smoking and Age.

Abbreviations; CI=Confidence Interval; LEEP; Loop Electrosurgical Excisional Procedure; N=numbers, OR=Odds Ratio

Table S2 The cervical microbiota six months post treatment in the LEEP group and the cervical microbiota in the Reference group.

|  | **Reference group**  **N=100** | | **LEEP, 6m follow-up**  **N=77** | | | **Unadjusted^1^** | | | **Adjusted^2^** | | |
| --- | --- | --- | --- | --- | --- | --- | --- | --- | --- | --- | --- |
| **Bacteria** | **n** | **%** | **n** | **%** | | **OR** | **CI** | **p** | **OR** | **CI** | **p** |
| *Bacteroides spp* | 0 | 0 | 2 | 3 | | **∞** |  | 0.19 | **∞** |  | 0.28 |
| *Chlamydia trachomatis* | 1 | 1 | 0 | 0 | |  |  |  |  |  |  |
| *Escherichia.coli* | 9 | 9 | 2 | 3 | | 0.3 | 0.1-1.3 | 0.12 | 0.2 | 0.04-1.3 | 0.09 |
| *Gardnerella vaginalis* | 9 | 9 | 21 | 27 | | 3.8 | 1.6-8.9 | 0.002 | 2.6 | 0.9-7.0 | 0.07 |
| *Mycoplasma hominis* | 3 | 3 | 10 | 13 | | 4.8 | 1.3-18.2 | 0.02 | 1.1 | 0.2-5.3 | 0.89 |
| *Streptococcus* spp | 10 | 10 | 2 | 3 | | 0.2 | 0.1-1.1 | 0.07 | 0.2 | 0.03-1.5 | 0.12 |
| *Ureaplasma parvum* | 16 | 16 | 25 | 32 | | 2.5 | 1.2-5.2 | 0.01 | 1.6 | 0.7-4.0 | 0.29 |
| *Ureaplasma urealyticum* | 3 | 3 | 3 | 4 | | 1.3 | 0.3-6.7 | 1.00 | 1.1 | 0.2-7.8 | 0.89 |
| *Lactobacillus* spp | 39 | 39 | 36 | 47 | | 1.4 | 0.8-2.5 | 0.36 | 1.0 | 0.5-2.0 | 1.00 |
|  | | | | | | | | | | | |
| any non-*Lactobacillus* | 41 | 41 | 44 | | 57 | 1.9 | 1.1-3.5 | 0.04 | 1.4 | 0.7-2.8 | 0.42 |

^1^ Fisher`s exact test

^2^ Logistic regression adjusted for Marital status, Hormonal Contraceptive, Smoking and Age; 176 cases analysed, 1 missing case in reference group because of missing data on smoking

Abbreviations; CI=Confidence Interval; LEEP; Loop Electrosurgical Excisional Procedure; m=months; N=numbers, OR=Odds Ratio

Table S3 The cervical microbiota 12 months post treatment in the LEEP group and the cervical microbiota in the Reference group.

|  | **Reference group**  **N=100** | | **LEEP 12m follow-up**  **N=72** | | **Unadjusted^1^** | | | **Adjusted^2^** | | |
| --- | --- | --- | --- | --- | --- | --- | --- | --- | --- | --- |
| **Bacteria** | **n** | **%** | **n** | **%** | **OR** | **CI** | **p** | **OR** | **CI** | **p** |
| *Bacteroides* spp | 0 | 0 | 3 | 4 | **∞** |  | .07 | **∞** |  | 0.18 |
| *Chlamydia trachomatis* | 1 | 1 | 0 | 0 |  |  |  |  |  |  |
| *Escherichia.coli* | 9 | 9 | 2 | 3 | 0.3 | 0.06-1.4 | 0.12 | 0.2 | 0.03-1.1 | 0.06 |
| *Gardnerella vaginalis* | 9 | 9 | 20 | 28 | 3.9 | 1.7-9.2 | 0.002 | 2.3 | 0.9-6.2 | 0.10 |
| *Mycoplasma hominis* | 3 | 3 | 8 | 11 | 4.0 | 1.0-15.8 | 0.05 | 1.4 | 0.3-6.8 | 0.69 |
| *Streptococcus* spp | 10 | 10 | 3 | 4 | 0.4 | 0.1-1.5 | 0.24 | 0.4 | 0.1-1.9 | 0.25 |
| *Ureaplasma parvum* | 16 | 16 | 26 | 36 | 3.0 | 1.5-6.1 | 0.004 | 1.9 | 0.8-4.5 | 0.15 |
| *Ureaplasma urealyticum* | 3 | 3 | 4 | 6 | 1.9 | 0.4-8.8 | 0.45 | 1.2 | 0.2-7.2 | 0.86 |
| *Lactobacillus* spp | 39 | 39 | 39 | 54 | 1.8 | 1.0-3.4 | 0.06 | 2.2 | 1.1-4.6 | 0.03 |
|  | | | | | | | | | | |
| any non-*Lactobacillus* | 41 | 41 | 39 | 54 | 1.7 | 0.9-3.1 | 0.09 | 1.2 | 0.6-2.6 | 0.58 |

^1^ Fisher`s exact test

^2^ Logistic regression adjusted for Marital status, Hormonal Contraceptive, Smoking and Age; 171 cases analysed, 1 missing case in reference group because of missing data on smoking

Abbreviations; CI=Confidence Interval; LEEP; Loop Electrosurgical Excisional Procedure; m=months; N=numbers, OR=Odds Ratio
